# Development of a short form of the Swedish version of the Hemophilia Activities List

**DOI:** 10.1038/s41598-025-02110-y

**Published:** 2025-07-07

**Authors:** Elisabeth Brodin, Katharina S. Sunnerhagen, Åsa Lundgren Nilsson

**Affiliations:** 1 Institute of Neuroscience and Physiology, The Sahlgrenska Academy, University of Gothenburg, Vita Stråket 12, fl. 4, 413 46 Gothenburg, Sweden; 2 Neurocare, Sahlgrenska University Hospital, Gothenburg, Sweden

**Keywords:** Hemophilia, Activities, Participation, Questionnaire, Patient-reported outcome, Health care, Haematological diseases, Outcomes research

## Abstract

An evaluation of an individual’s function, activity, and participation in everyday life is an important component of long-term follow-up in health care. The Hemophilia Activities List (HAL) is an instrument that focuses on self-assessed activities and participation in individuals. The original HAL included 42 questions, many of which covered themes that overlapped with one another. To reduce the length of this questionnaire, data collected from the HAL from Swedish persons with hemophilia were analyzed using a three-step process including Rasch modeling, an equidiscriminatory item-total correlation approach, and patient views. Results obtained were combined to generate a 14-item questionnaire. Of note, although it was not considered a separate entity, the function of the upper extremities was addressed as a component of several other domains. The 14-item short form yielded median scores of 85.5 (range 11–100), which were similar to those achieved using the original 42-item version (median score 83, range 13–100). The short form can be used to complement other self-assessment instruments as part of an annual follow-up and will be particularly helpful to identify issues and follow activity and participation over time.

## Introduction

Hemophilia is a recessive X-linked hereditary disorder characterized by an increased tendency to bleed. The two main types of this disorder are hemophilia A, which results from a deficiency of clotting factor VIII, and hemophilia B, which is a deficiency of clotting factor IX^1^. Hemophilia can be classified as severe (< 1% of normal factor activity), moderate (1–5%), or mild (6–40%)^2^. In Sweden, there are ~ 1000 males (including children) diagnosed with hemophilia, with hemophilia A identified more frequently (~ 80%) than hemophilia B (~ 20%). Approximately 50% of the PWHs (persons with hemophilia) have either the moderate or the severe form of the disease^3^. Clinical/objective observations must be combined with information from self-reported instruments to capture the entire spectrum of the disease^4^.

Many young adults who have received appropriate prophylaxis (i.e., treatment with missing coagulation factors) have not developed the physical impairments associated with the recurrent joint and muscle bleeds typically experienced by older PWHs. This has led to a ceiling effect in the HAL questionnaire which limits differentiation among those with mild symptoms regardless of the person’s age^5^. While older PWHs may have more significant disabilities and thus reduced activity and participation in everyday life, their conditions vary substantially; some older PWHs present with domain scores indicating activities and abilities at near-normal levels^6^. The most common arthropathy is in the weight bearing joints therefore are activities involving knees and ankles more problematic for PWH together with the elbow^7^.

Validated instruments and assessments will be needed to evaluate individual performance as well as the most effective forms of intervention in this disease. Because interventions are often specific to individual countries, regions, and/or clinics, the evaluation tools available also vary substantially. This has led to an increased need for appropriately designed comparison studies^8^.

PWHs are routinely asked to provide opinions and insights, particularly those that highlight the impact of the disease and its treatment on health-related quality of life, anxiety, pain, and physical activity. This information is used in conjunction with other measurements, including joint status and laboratory data^9–12^. Several validity and reliability studies of patient-reported outcomes have been published^13–18^. One published review that focused on physical activity in PWHs highlighted the limited evidence for a link between bleeding phenotype and physical activity^19^. However, the authors of this report found evidence for improvements in treatment over time that provided PWHs with the opportunity to be more physically active^19^.

The Hemophilia Activities List (HAL) is a questionnaire that was originally developed for use in the Netherlands. It includes 42 questions that are divided into seven domains with three complex domains based on the disability model presented by the International Classification of Functioning, Disability, and Health (ICF)^20^. The items addressed in the HAL overlap considerably with one another^15,21^. Thus, it may be possible to reduce the number of questions and limit the administrative burden associated with this questionnaire for all concerned.

In a parallel study, a short (18-question) form of the HAL was developed by its originators in the Netherlands that was based on data from a patient cohort from the United States^22^. This short form was validated with the 36-Item Short Form Health Survey (SF-36) and the EuroQol 5 Dimension 5 (EQ5D).

Overall it is important that evaluation tools such as questionnaires are validated in the context that they are used as the conditions for healthcare and social life are different in different countries^23^. A review concludes that further research needs to focus on measurement error, responsiveness, interpretability, cross-cultural validity of the self-reported tools measuring activities in PWHs^24^. A new questionnaire, ACTIVLIM-Hemo, which is linear, has been developed recently. It is a valid and reliable alternative to HAL in assessing activity limitations in PWH^25^.

This study aimed to develop a short form of the Swedish-validated version of the HAL based on a Swedish cohort of PWHs. Our goal was to minimize the administrative burden associated with this questionnaire to be completed by PWHs during their annual visits to the clinic.

## Results

### Combining the results

**Fig. 1:**
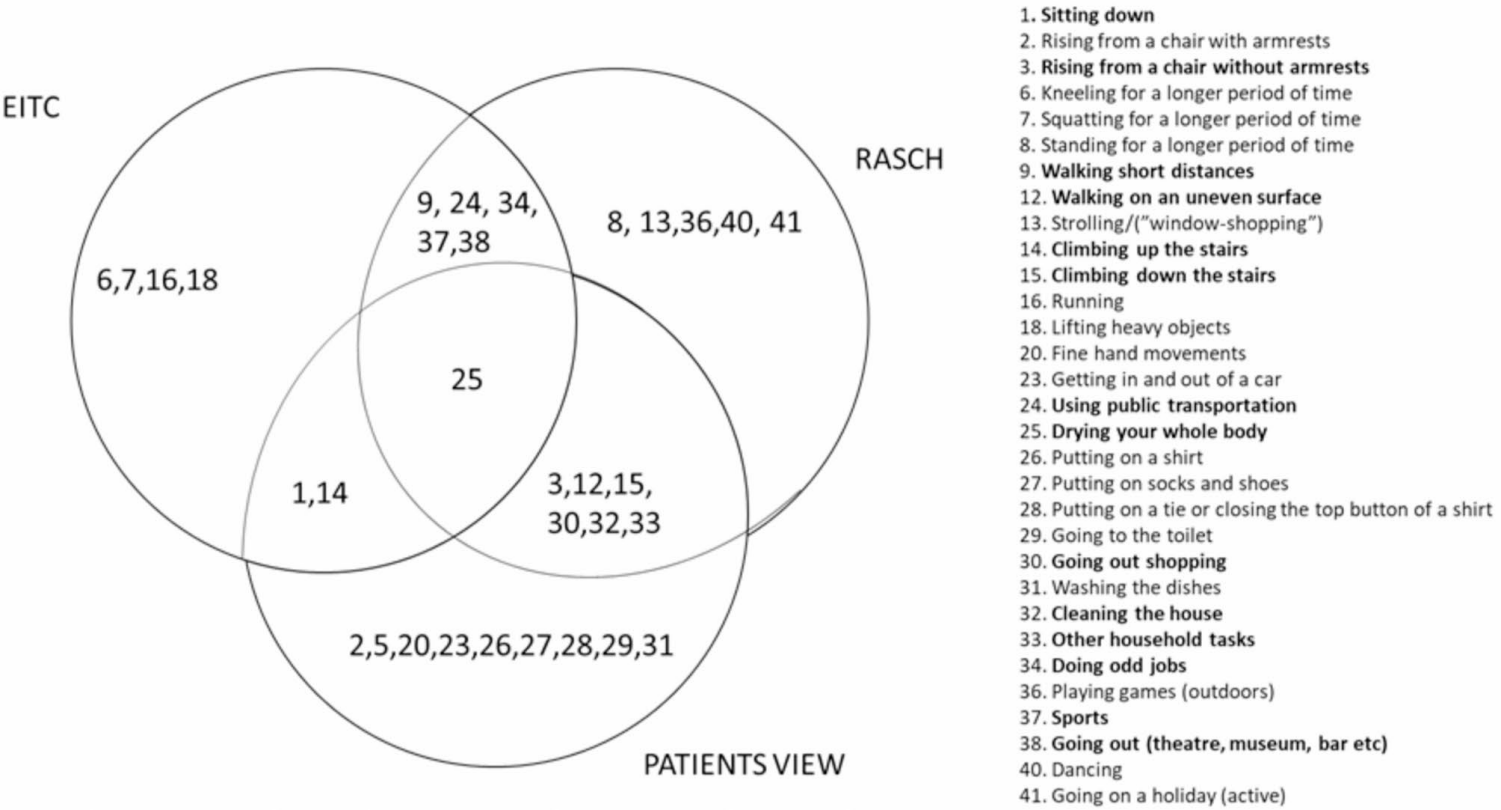
Results from three different methods were merged to generate 14 specific questions to include in the Swedish short version of the Hemophilia Activities List (HAL). Please see Table 1 for additional information. EITC (equidiscriminatory item-total correlation).

We combined the results from three different methods to generate a 14-item short form of the Swedish version of the HAL (Fig. 1 and Table 1). If the question is included in the results of two of the three statistical calculations, it was included in the final version of the short form of HAL.

**Table 1:** The results from the three steps (Fig. 1) were merged to create a Swedish short form of the HAL.

HAL domains	Rasch	EITC	Patiens view	Swedish HAL, short form
“lyingdown/sitting/kneeling/standing”	**3.** 8.	**1.** 6. 7.	**1.** 2. **3.** 5.	*1.* *3.*
“function of the legs”	**9.** **12.** 13. **15.**	**9.** **14.** 16.	**12.** **14.** **15.**	*9.* *12.* *14.* *15.*
“function of the arms”	–	18.	20.	–
“use of transportation”	**24.**	**24.**	23.	*24.*
“self-care”	**25.**	**25.**	**25.** 26. 27. 28. 29.	*25.*
“household tasks”	**30.** **32.** **33.** **34.**	**34.**	**30.** 31. **32.** **33.**	*30.* *32.* *33.* *34.*
“leisure activities and sports”	36. **37.** **38.** 40. 41.	**37.** **38.**	_	*37.* *38.*

Shown is the 3 different methods and a summary of the 14 items included in the final short form of the Swedish HAL. The bold numbers are the questions that remained in the Swedish short form of HAL.

Significant values are in (italics).

This short form included six of the original seven domains; the domain that considered upper extremity function was not represented. Of note, domain-limited calculations cannot be performed when using the short form because only a few items were included in each category.

The 14 questions included in the short-form questionnaire address several critical ICF categories, including changing body position (d410; n = 2), walking (d450; n = 4), using transportation (d470; n = 1), self-care (d5; n = 1), domestic life (d6; n = 4), and sports and leisure activities (d920; n = 2)^26^.

Correlations of normalized scores (0–100) of the 14 items identified by the 128 PWHs who evaluated the 42 items included in the original full-length HAL resulted in a Spearman r = 0.964 (significance level at 0.01). An increasing monotonic trend between short form of HAL and original HAL. This means that selected questions in the suggested short form of HAL relate almost linearly to the original HAL with 42 questions. The median score of these 14 items was 85.5 (range, 10–100 with 0 and 100 as the lowest and highest possible scores, respectively). The median score of the original 42 items was 83 (range 13–100).

The results of the Rasch analysis revealed that two of the items included in the final version of the Swedish short HAL (numbers 1 and 14) were disordered.

### The Rasch approach

The results of the Rasch analysis revealed that 18 of the original 42 questions^21^ with no disordering represented some of the more complex activities (Table 1). To obtain a questionnaire with fewer items that nonetheless represented the overall HAL, we began with these 42 items. Of note, the Rasch analysis revealed some degree of local dependency. While this analysis also revealed frequent disordering between the items, none were identified as misfits. While the Rasch analysis identified 17 items that had no disordering or misfit, local dependency was detected frequently in all items in the Swedish HAL. These items address several of the more complex activities associated with upper and lower extremity functions. The 17 items included in this short version of the Swedish HAL included two from the lying down/sitting/kneeling/standing domain, four from the functions of the leg domain, none from the functions of the arms domain, one from the use of transportation domain, one from the self-care domain, four from the household tasks domain, and five from the leisure activities and sports domain. Because these items overlap with one another (for example, the complex activities depend directly on the function of the joints and muscles of both the arms and legs) and the small cohort used to generate these findings, the goal of no local dependency could not be achieved in this study.

### The equidiscriminatory item-total correlation (EITC) approach

Twelve questions were selected using the EITC approach, including one from each domain and each percentile (Table 1). The same four questions were selected in each of the three percentiles. The correlation coefficients for the different questions varied from 0.765 to 0.969 in the three different percentiles.

### The patients’ views

Sixteen of 30 PWHs responded to our invitation to participate in the study. Of this group, two responded that they would not be participating in the study, and one presented with a language difficulty and was unable to answer the questionnaire. Of the 13 potential participants remaining, 10 who had provided their telephone contact information were interviewed about how they thought HAL might be used and if they had suggestions on other topics related to hemophilia. All 13 of these participants also evaluated all 42 questions on the original HAL and indicated the importance of each activity to their hemophilia-related dysfunction as “very important”, “important”, or “less important”^27^. A question was retained if 8 or more of the 13 participants (> 61%) described the question as important.

The PWHs expressed their opinion of the HAL questionnaire as follows: “…since I have no problems with my hemophilia, I got stuck in a response voice when I answered and the answers were the same for all the questions…”; “…I don´t think anything is missing…”; “…too many questions, too long…”; “…some activities not relevant for me due to my age and joint problems…”; and “…can be used for follow-up not only at annual check-ups but also before and after treatment/intervention…”. Some PWHs suggested that the HAL might be used to complement the annual joint survey; others noted that they would like to have the opportunity to write about a personal activity that might be followed up. The summarized patient view was that the HAL questionnaire was too long and that when one has no hemophilia-related joint problems, all the answers are the same (i.e., either not important or very important, depending on the structure of the question). The responses of PWHs with joint-related problems were more variable.

As a result of our analysis of patient views, 18 items were retained (Table 1).

## Discussion

This study presents an abbreviated version of the HAL that can be made available for use by PWHs and their healthcare providers in Sweden. By merging the results from three different methods, the length of the questionnaire was reduced from 42 to 14 items. This abbreviated version could reduce the burden on the patient who typically needs to complete several forms that measure both health status and the outcomes of their prophylaxis and treatment regimens. A short form of HAL takes less time to respond, and overlaps are reduced, which can motivate patients to respond to the form to a greater extent.

All 42 questions in the original long-form HAL describe activities that rely directly on joint function. Thus, it was not surprising that the results of the Rasch analysis included numerous examples of local dependency. Similarly, disordered items were detected frequently, albeit not universally. The merged short form of the HAL included three disordered items. These items were retained because they were included in the scales of the results obtained from the other two analysis methods.

It will be of great interest, both scientifically and clinically, to be able to evaluate interventions based on rehabilitation strategies. To achieve this goal, instruments with the same structural characteristics will be needed to evaluate outcomes in different situations. Questionnaires might also be used to identify patients’ self-perceived abilities and promote open comparisons between clinics.

The original 42-item HAL exhibited a pronounced ceiling effect, most notably among the younger PWHs^5^. Interestingly, the inter-item correlations in the original version of the HAL were very high, with a Cronbach alpha of 0.89 –0.98 except for the items included within the transportation domain (Cronbach alpha = 0.71)^15^. In contrast to previous decades, today the care of PWHs focuses on their own perceptions and experiences and their ability to be active and participate in society like anyone else. This approach has created numerous evaluation instruments, primarily in the form of questionnaires. These questionnaires must be designed to measure the desired phenomena without too much repetition, but at the same time, they must ensure that all topics of importance to this cohort are included and addressed^28^.

All questionnaires presented to PWHs at their yearly clinic visit must provide an adequate view of their health and well-being as well as an assessment that is appropriate for use by the health care provider. The questionnaire must be perceived as sufficiently meaningful to justify the time needed for its completion. It is also important to cover all aspects of a given topic. The 14 items included in the short version of the HAL presented in this study were generated using the three steps described; six of the original seven domains were represented. The only domain that was not represented in this 14-item questionnaire is the upper extremity function. However, this function is clearly reflected in some of the more complex items included in the self-care and domestic life domains. This will ensure that important content related to activity and participation is addressed; the overall validity determined by an investigator assessment confirmed this point.

We compared our findings with the 18-item short form of the HAL developed by a group in the Netherlands based on findings from a patient cohort from the United States. Interestingly, we found that eight of the questions included in our version are the same as those featured in this earlier version^22^. We also compared the median value obtained in our 14-item short form (85.5, range 10–100) to that reported in the earlier 18-item study (83.5, range 13–100); our analysis revealed a Spearman’s correlation coefficient of 0.954 at the 0.01 significance level. This result indicates that either version might be used to assess PWH in Sweden. However, the 18-item version from the Netherlands includes 10 disordered items when applied to PWHs in Sweden.

The items covered in ICF chapters d4, d5, d6, and d9 capture both the activities of PWHs as well as their participation in society. Most of the questions included in our short form HAL focus on participation. Self-assessment is an important parameter to consider when evaluating the medical regimen used to treat PWHs and should be considered complementary to laboratory tests, joint status, and radiographic responses. Similar to the results from a study from the United States, many PWHs responded to questions within the “use of transportation” domain as “not applicable”^14^. While this domain may not be as applicable today as it was previously, it continues to symbolize an important form of participation in everyday life. We note that the median normalized score of the 42 items included in the full HAL is similar to that of the 14 items in our abbreviated version. Thus, the short form of the HAL should be sufficient to capture the activity and participation of PWHs as effectively as the original.

One limitation of this research is the small size of the study population; only 128 PWHs were evaluated by Rasch analysis. While there are approximately twice as many adults with moderate or severe hemophilia in Sweden, it is difficult to organize a total population study. Not all PWHs, who met the inclusion criteria, agreed to participate in the study. The dropout rate was about 50%. You don´t know anything about them who don´t participate. Also, the population that would be required for statistical significance is about 6 times more than what is available in Sweden.

Furthermore, because we did not include participants with mild hemophilia in this study, it is not possible to comment on the use of the abbreviated Swedish HAL to evaluate their activity and participation at this time. Of note, the original Swedish HAL was also validated for moderate and severe hemophilia and did not address mild disease^15^. This may be because persons with mild hemophilia do not typically develop bleeding in joints and muscles and thus do not experience many of these hemophilia-associated physical problems in everyday life. In the interview part, the population was limited to one of three clinics in Sweden. Living circumstances can therefore differ depending on where you are registered.

The strength of this study is that the short version of the HAL was developed by merging the results of two statistical methods with an analysis of patient views. The PWHs interviewed for this study exhibited a wide range of ages and musculoskeletal problems and thus covered the spectrum of conditions leading to diminished activity and participation.

The next step will be to test the 14-item short form of the Swedish HAL in a population study to determine its applicability for assessing the activity and participation of patients with moderate to severe hemophilia currently living in Sweden. It is important to verify that the 14-item short form retains the validity and reliability of the original 42-item long form. The results obtained using the short form developed in the Netherlands were in good agreement with the full version of the HAL^29^. The assessment burden on PWHs is then reduced without losing the quality of the information. The short form will be easier to complete and will facilitate more rapid evaluations by the clinician. It might also be interesting to compare the results of the short form developed in the Netherlands^22^ with those obtained using the Swedish short version in a new population study.

It would also be desirable to validate the Swedish short form of HAL against other questionnaires used in research and clinics around the world to compare the results of given care. However, it requires that new questionnaires will be validated according to good research ethics through the forward backward methodology before this comparison can be made.

ACTIVELIM-Hemo is another new scale that was recently developed in Belgium. This instrument includes 22 activities that are ranked in three scale steps^25,30^. The authors found this scale to be more useful than HAL for following activity levels of PWH, especially those related to treatment. ACTIVELIM-Hemo has a smaller ceiling effect than the HAL and a unidimensional and linear scale based on its creation using a Rasch rating scale. A direct comparison between the Swedish short HAL and ACTIVELIM-Hemo could also provide useful insights and information. This is especially the case for the younger PWHs who were provided with modern treatment methods beginning in childhood, and who are expected to maintain activity levels similar to those of the normal population. First, ACTIVLIM-Hemo must be validated for the Swedish PWHs.

The study was supported in part by an unrestricted grant from the Swedish Orphan Biovitrum (Sobi) to EB. The study was also financed by grants from the Swedish state under an agreement between the Swedish government and the county councils, the ALF agreement (ALF Gbg-965653 to KSS).

## Material and methods

Responses to all questions included in the HAL are rated on a six-point Likert scale. The sum of the final HAL score is normalized on a scale from 0–100, with 0 representing the lowest and 100 the highest possible functional abilities^21^. The HAL addresses seven specific domains, including (1) lying/sitting/kneeling/standing; (2) functions of the legs; (3) functions of the arms; (4) use of transportation; (5) self-care; (6) household tasks; and (7) leisure activities and sports. The three combined domains are (1) upper extremity activities; (2) basic lower extremity activities; and (3) complex lower extremity activities^21^.

Three specific techniques were used to reduce the number of items addressed, including two statistical methods: item response theory (Rasch modeling, RUMM laboratory^®^) and equidiscriminatory item-total correlation (EITC)^31^ The findings obtained using these methods were merged with the results of interviews in which PWHs were asked to identify the least and most important activity items, consistent with concepts addressed by the original HAL.

This approach was inspired by the methods used to develop QuickDASH^32^. Three different scales were created; the final scale was a combination of these three approaches in which at least two methods represented the selected item.

### Cohort data

We performed two separate analyses of results from a previously published follow-up study that used the full Swedish version of the HAL^5^. Of the 128 patients included in this study, 99 were diagnosed with hemophilia A and 29 with hemophilia B; of these patients, 31 had moderate and 97 had the severe form of the disease. Most of the participants (n = 102) received some form of routine prophylaxis.

Patient perspectives were included in both the questionnaires and interviews. PWHs were asked to identify which of the 42 questions provided important insight into their ability to participate in everyday life and to function as active members of society. Thirteen PWHs representing different ages and hemophilia severity who were followed by the Coagulation Center at Sahlgrens University Hospital participated in this study and rated the 42 questions as very important, important, or less important.

In the follow-up study, patients were divided into two groups and followed over time. One for those with early prophylaxis treatment and one for patients where treatment started later in life. Results from a study using the Elisabeth Svensson method^33^ showed that younger people with early treatment had a ceiling effect with HAL, while older people showed different results depending on physical limitations. Thus, it can be predicted that age and when in life prophylaxis treatment starts affecting responses to HAL and are confounding factors^5^. Neither that study nor this one has included PWH with mild form, so it is not possible to comment on the disease severity.

### Rasch modeling

The item reduction techniques featured in this study were based on item response theory (Rasch modeling). RUMM 2030 (RUMM laboratory^®^) was used to identify misfits, disordered responses, and local dependency on a question-by-question basis. Our goal was to identify the most valuable and useful questions for self-assessed activity and participation by PWHs.

The Rasch model focuses on the relative severity of the questions and the relative ability of the respondents. The resulting calibrations and measurements, expressed in units of measure at equal intervals, are based on the logit underlying a given skill point, i.e., when a person with a given ability is observed performing a specific activity. This scale should apply in the same way regardless of the age, gender, and disease subtype of the group under evaluation^34^. In other words, this type of analysis indicates how well the items in question and the persons assessed fit the model and suggests items that might be rejected^35^.

Selected items should not be disordered or have local dependency or misfit. This analysis was performed for all 42 items included in the original HAL. Various response options were analyzed to determine whether all alternative answers were employed. The overall goal was to identify items that represented activities in the absence of disordering, local dependency, or misfit, representing all domains in HAL.

### The equidiscriminatory item-total correlation (EITC) approach

Item reduction was performed using an EITC approach by selecting the items with the highest correlation of the sum score at the 25th, 50th, and 75th percentiles^31^. For example, the distribution of PWHs below the 25th percentile score was given a score of zero, while those at or above this percentile were given a score of one. The correlation between the sum score and the new item score (i.e., one or zero) was calculated and the items were then ranked in order of their item-total correlation. This approach provided us with three lists of items corresponding to the 25th, 50th, and 75th percentiles^36^.The two questions with the highest correlations of the sum scores in each of the seven domains were selected from each of the three percentiles. The top-ranked item in each domain was then chosen. If the same question was ranked first or second in all percentiles, the second question was not included. This approach permitted us to obtain reliable distinctions at all levels.

### The patients’ views

We selected 30 patients diagnosed with either moderate or severe hemophilia A or B who were followed at the Coagulation Center in Gothenburg and invited them by mail to participate in this study. The patients in this group varied in age and hemophilia severity and presented with different disease-related symptoms. The PWHs were asked to evaluate each of the 42 questions included in the full HAL and to score them as “very important”, “important”, or “less important” for describing how hemophilia affected their activity and participation in society. Each PWH was then invited to participate in a telephone interview about the HAL questionnaire to share anything else they might want to communicate or suggest regarding the use of the HAL questionnaire. The information provided by the PWHs was then analyzed; the responses listed as “important” and “very important” were combined into a single group. Questions were retained if ≥ 60% of the PWHs designated them as “important” or “very important”.

### The merge steps

The results obtained using the three different methods were merged into a final short version of the Swedish HAL questionnaire. Items that were marked as important or very important in at least two of the three component methods were retained, similar to methods used in the aforementioned QuickDASH study^32^.

## Conclusion

In conclusion, the Swedish short form of HAL presented in this study includes 14 items that address six of the original seven domains. This new version focuses more on participation than activity and may become the preferred self-assessment form for the annual clinic check-ups as well as the evaluation of acute and chronic interventions.

## Data Availability

Due to the sensitivie content, the datasets generated and analysed during this study are available from the corresponding author on reasonable request.
